# MicroRNA and histopathological characterization of pure mucinous breast carcinoma

**DOI:** 10.7497/j.issn.2095-3941.2013.01.004

**Published:** 2013-03

**Authors:** Feng Zhou, Shuai Li, Hui-Min Meng, Li-Qiang Qi, Lin Gu

**Affiliations:** 1 Departments of Breast Oncology/Surgery, Tianjin Medical University Cancer Institute and Hospital; Key Laboratory of Breast Cancer Prevention and Therapy, Ministry of Education; Key Laboratory of Cancer Prevention and Therapy, Tianjin; State Key Laboratory of Breast Cancer Research, Tianjin 300060, China; 2 Department of Breast Cancer Pathology and Research Laboratory, Tianjin Medical University Cancer Institute and Hospital; Key Laboratory of Breast Cancer Prevention and Therapy, Ministry of Education; Key Laboratory of Cancer Prevention and Therapy, Tianjin; State Key Laboratory of Breast Cancer Research, Tianjin 300060, China

**Keywords:** Pure mucinous breast carcinoma, microRNA, real-time PCR

## Abstract

**Objective:**

Pure mucinous breast carcinoma (PMBC) is an uncommon histological type of breast cancer characterized by a large amount of mucin production. MicroRNA (miRNA) is a large class of small noncoding RNA of about 22 nt involved in the regulation of various biological processes. This study aims to identify the miRNA expression profile in PMBC.

**Methods:**

MiRNA expression profiles in 11 PMBCs were analyzed by miRNA-microarray and real-time polymerase chain reaction (PCR). Thirty-one PMBCs and 27 invasive ductal carcinoma of no special types (IDC-NSTs) were assessed by immunohistochemistry using antibodies against ER, PR-progesterone receptor, HER2, Ki-67, Bcl-2, p53, PCNA, and CK5 and 6.

**Results:**

We analyzed the miRNA expression in 11 PMBCs and corresponding normal tissues using miRNA-microarray and real-time PCR, and found that miR-143 and miR-224-5p were significantly downregulated in mucinous carcinoma tissue. Compared with IDC-NSTs, PMBC showed a significantly higher ER positive rate, lower HER-2 positive rate, and lower cell proliferation rates.

**Conclusions:**

To our knowledge, this is the first study to demonstrate the miRNA expression profile of PMBC, and our findings may lead to further understanding of this type of breast cancer.

## Introduction

Pure mucinous breast carcinoma (PMBC) is a rare variant of breast cancer characterized by abundant production of extracellular and/or intracellular mucin. PMBC accounts for about 2% of all primary breast cancers and is usually associated with a better clinical outcome than invasive ductal carcinoma^1^^-^^4^. PMBC mostly affects elder women, and only 1% of PMBC patients is below 35 years old^2^^,^^5^.

MiRNA is a novel class of short noncoding RNA that has a significant role in gene regulation^6^. MiRNA controls cell growth, proliferation, metabolism, and apoptosis by binding to the 3’-untranslated region (3’-UTR) of target Mrna^6^^,^^7^. More than half of known human miRNA genes are located in chromosomal fragile sites that are susceptible to structural genomic alterations during tumor development^8^. Specific miRNA dysregulation has been shown to correlate with particular types of cancer, and miRNA expression profiles could distinguish different cancer types^9^^,^^10^. For example, miR-10b, which is overexpressed in metastatic breast cancer cell lines, can suppress HOX10D^11^, whereas miR-21, which is upregulated in many kinds of cancers, can inhibit the expression of PDCD4^12^.

We analyzed the miRNA expression profile of 11 PMBCs and corresponding normal tissues and found that miR-143 and miR-224-5p were significantly downregulated in PMBC. We also analyzed the immunohistochemical features of 31 PMBCs and 27 IDC-NSTs. Our findings may help us further understand PMBC.

## Materials and methods

### Clinical data

**Figure 1:**
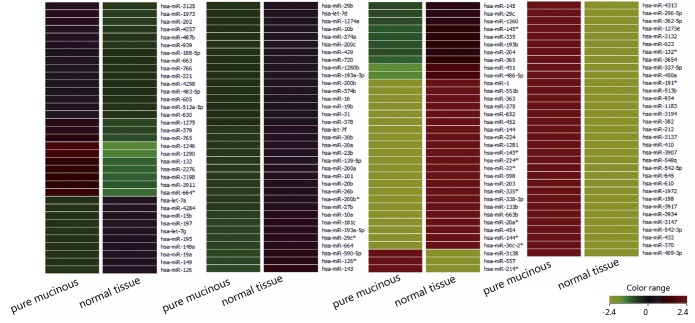
MicroRNA expression profiling of PMBC tissue and corresponding normal tissues. MicroRNAs were detected by microarray. A heat map was generated from the average of normalized log-transformed fluorescent intensity for each data set.

Tissue samples of 11 PMBCs and corresponding normal tissues were obtained from patients who underwent breast cancer surgery (**Table 1**). Two microarray hybridization studies were performed on a pair of PMBC-derived and matching adjacent normal tissue-derived RNA (**Figure 1**). To validate the miRNA-array results in a larger series of patients, we employed real-time PCR to analyze the expression of miRNA in 10 other PMBCs.

**Table 1:** Patient clinical features

No.	Age	Tumor size (cm)	Histology grade	Lymph node status	ER (%)	PR (%)	HER-2	Normalized miRNA amount in mucinous carcinoma tissue relative to adjacent normal tissue 2^-△△Ct^				
								miR-143	miR-203	miR-224-5p	miR-375	miR-451a
1	77	2.2	pT_2_N_0_M_x_	0/8	90	80	-	2.4538	11.8762	4.0278	155.9565	404.5012
2	62	2.5	pT_2_N_0_M_x_	0/16	90	90	-	0.0016	0.0021	0.0003	0.0002	0.0018
3	61	2.1	pT_2_N_0_M_x_	0/18	70	60	-	0.1539	5.2598	0.0437	2.8580	10.0910
4	47	2.2	pT_2_N_1_M_x_	1/20	90	<1	-	0.2230	0.4649	1.6760	8.7847	1.6994
5	53	5.5	pT_3_N_1_M_x_	3/19	90	15	+	0.0234	0.3475	0.0018	0.3560	0.0042
6	57	4.5	pT_2_N_0_M_x_	0/23	95	80	-	0.3572	2.7416	0.9760	7.8354	0.3451
7	62	2.5	pT_2_N_0_M_x_	0/12	90	-	-	2.0634	1.4743	0.9428	35.1390	0.6285
8	60	1.8	pT_1_cN_0_M_x_	0/20	80	10	-	0.0036	0.1005	0.0008	0.0321	0.0067
9	61	3.2	pT_2_N_1_M_x_	1/19	15	-	-	0.0554	0.5087	0.0799	0.2415	0.1451
10	63	1.5	pT_1_N_0_M_x_	0/10	70	50	-	0.0005	0.0079	0.0003	0.1001	0.0027

Relative quantification was performed by the 2^-△△Ct^ method with adjacent normal tissue as a calibrator. Every independent analysis was carried out after the RNA extraction step. Total RNA was Poly-A tailed, reverse transcripted, and then real-time PCR tested. △C_T_ obtained from real-time PCR was subject to matched Wilcoxon rank sum test (△C_T_=C_T miR_–C_T U6_). miR-143, *P*=0.013, Z=-2.497; miR-203, *P*=0.386, Z=-0.866; miR-224-5p, *P*=0.037, Z=-2.090; miR-375, *P*=0.959, Z=-0.051; miR-451, *P*=0.241, Z=-1.172.

Written informed consent was obtained from all patients, and the study was approved by the institutional review board. PMBCs and corresponding normal tissues were collected from breast cancer surgical specimens and stored at Tianjin Medical University Cancer Institute and Hospital. No patient included in this study received radiation therapy or chemotherapy before the surgery. Fresh samples were frozen shortly after resection and stored at -80 °C.

### RNA extraction and RT-qPCR for miRNA

Total RNA was isolated using Trizol (Invitrogen, Carlsbad, CA) from freshly frozen tissues following the manufacturers’ protocols. Total RNA was poly-A tailed, reverse transcribed, and analyzed on CFX96 (BioRad), as previously described^13^. MiRNA expression was normalized to U6. The ΔCt (ΔCt=Ct_miRNA_-Ct_U6_) obtained from the PMBCs and the corresponding normal tissues were compared using the matched Wilcoxon rank sum test.

### Immunohistochemistry

About 4 μm thick formalin-fixed, paraffin-embedded tissues were dewaxed, hydrated, heated for 2 min in a conventional pressure cooler (antigen retrieval), treated with 3% H_2_O_2_ for 10 min (reduction of endogenous activity), and then incubated with normal goat serum for 10 min (elimination nonspecific staining). The sections were incubated at 37 °C with antibodies, including ER (mouse IgG, Zymed, USA), PR (mouse IgG, Zymed, USA), HER-2 (mouse IgG, Newmarkers, USA), Ki-67 (mouse IgG, Zymed, USA), Bcl-2 (mouse IgG, ZSGB-Bio, China), p53 (mouse IgG, Zymed, USA), PCNA (mouse IgG, ZSGB-Bio, China) and CK5 and 6 (mouse IgG, ZSGB-Bio, China). After washing, the sections were incubated with biotin-labeled secondary antibody against mouse immunoglobulin for 20 min at room temperature. Then, the slides were rinsed and covered with streptavidin-biotin-peroxidase for 20 min. All sections were counterstained with 3,3’-diaminobenzidine tetrahydrochloride. Slides were counterstained with hematoxylin and mounted for light microscopy.

## Results

### MiRNA expression profile in pure mucinous breast carcinoma

Results of the tests show that miR-143 and miR-224-5p were significantly downregulated in PMBC tissues compared with their corresponding normal tissues (**Table 1**).

MiR-143 and miR-224-5p have been reported to be dysregulated in many kinds of cancers, including prostate cancer, colon cancer, bladder cancer, renal cancer and hepatocellular carcinoma^14^^-^^19^. These two miRNAs were found to be downregulated in PMBC for the first time, thus helping us further understand the molecular mechanism underlying this type of breast cancer.

### Immunohistochemical analysis of PMBC

**Figure 2:**
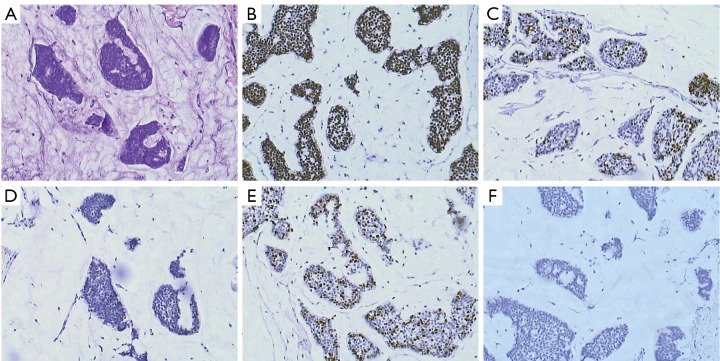
Immunohistochemical features of PMBC. Representative micrograph of a PMBC (A). H&E staining and immunohistochemical staining of ER (B), PR (C), HER-2 (D), Ki-67 (E), and p53 (F).

The histopathological and immunohistochemical features of PMBC are summarized in **Table 2**. The vast majority of PMBCs were of low cell proliferation rates (Ki-67 labeling), high ER positive rate (*n*=27/31), high PR positive rate (*n*=28/31), low HER-2 positive rate (*n*=0/31) and can be classified as luminal A according to the criteria of Nielsen *et al.*^20^. The representative micrographs of the immunohistochemical features of PMBC are shown in **Figure 2**.

**Table 2:** Histopathological and immunohistochemical features of 31 PMBC and 27 IDC-NSTs

	PMBC (n=31)	IDC-NSTs (n=27)	P value *
Lympho node invasion			0.050
Present	6 (19.3%)	12 (44.4%)	
Absent	25 (80.7%)	15 (55.6%)	
ER			<0.001
Positive	27 (87.1%)	11 (40.7%)	
Negative	4 (12.9%)	16 (59.3%)	
PR			0.164
Positive	28 (90.3%)	20 (74.1%)	
Negative	3 (9.7%)	7 (25.9%)	
HER-2			<0.001
Positive	0 (0%)	11 (40.7%)	
Negative	31 (100%)	16 (59.3%)	
Ki67			<0.001
Low (<10%)	18 (58.0%)	1 (3.7%)	
Intermediate	10 (32.3%)	14 (51.9%)	
High (>30%)	3 (9.7%)	12 (44.4%)	
Bcl-2			0.260
Positive	24 (77.4%)	17 (63.0%)	
Negative	7 (22.6%)	10 (37.0%)	
P53			0.091
Positive	3 (9.7%)	8 (29.6%)	
Negative	28 (90.3%)	19 (70.4%)	
PCNA			0.191
<19%	0 (0%)	2 (7.4%)	
20%~39%	9 (29.0%)	3 (11.1%)	
40%~61%	5 (16.1%)	5 (18.5%)	
≥62%	17 (54.8%)	17 (63.0%)	
CK5&6			0.593
Positive	11 (35.5%)	12 (44.4%)	
Negative	20 (64.5%)	15 (55.6%)	

*Chi-squared test. ER, oestrogen receptor; PR, progesterone receptor; PMBC, pure mucinous breast carcinoma; IDC-NST, invasive ductal carcinomas of no special type.

### Comparison of immunohistochemical features between PMBC and IDC-NST

PMBC showed significantly higher ER positive rate (*P*<0.001), lower HER-2 positive rate (*P*<0.001), and lower cell proliferation rate (Ki-67 labeling, *P*<0.001) compared with IDC-NSTs. The result of ER staining was consistent with the former immunohistochemical findings of other groups^2^^,^^3^. However, the Ki-67 labeling result was in direct conflict with the findings of Lacroix-Triki *et al.*^3^. Their study demonstrated that no difference in Ki-67 labeling rates existed between PMBC and IDC-NST. The conflict may be caused by different patient selections. Lacroix-Triki *et al.*^3^. chose ER-matched PMBC and IDC-NST patients in carrying out immunohistochemical staining.

## Discussion

PMBC is a rare type of invasive breast cancer characterized by cluster-arranged tumor cells floating in a large amount of mucin. PMBCs account for about 2% of all breast cancers and have a better prognosis than IDC-NSTs. PMBC is a disease common among elder women, and only 1% of PMBC patients are below 35 years old. MicroRNA is a novel class of small noncoding RNA, which is always deregulated in cancer and has a key role in cancer progression. In this study, we attempted to analyze the miRNA expression profile to uncover its role in PMBC. We explored the miRNA expression profile in PMBC tissues and corresponding normal tissues using miRNA-microarray and real-time PCR. MiR-143 and miR-224-5p were found to be significantly downregulated in PMBC. To our knowledge, this is the first study to analyze miRNA expression pattern in PMBC. Our findings may provide further understanding of PMBC.

About half of annotated human miRNA genes are located in chromosomal fragile sites or regions of the genome that are associated with cancer^8^. Aberrantly expressed miRNAs are associated with many types of cancers^9^^,^^10^. More importantly, miRNA expression patterns can be correlated with cancer type, stage, and other clinical variables, suggesting that miRNAs can function as novel biomarkers for cancer diagnosis^9^. MiR-143 and miR-224-5p have been reported to be dysregulated in many kinds of cancers, including prostate, colon, bladder, renal and hepatocellular carcinoma^14^^-^^18^. For example, miR-143 expression is reportedly downregulated in various human cancers^15^ and is regulated in many cancer-related genes including KRAS^21^, DNMT3A^22^, MYO6^23^, Bcl-2^24^ and ERK5^25^. Borralho *et al.*^15^ showed that miR-143 overexpression can impair colon carcinoma xenograft growth in mice, induce cell proliferation, and inhibit cell apoptosis. Thus miR-143 is a pivotal regulator of gene expression in cancer tissues. Moreover, Chang *et al.*^26^ found that reduced expression of miR-143 is associated with aggressive mucinous phenotypes in colorectal cancer. This finding indicates that the downregulation of miR-143 may be a common event in the formation of mucinous cancer phenotype.

PMBC has higher ER expression rates (*P*<0.001) and lower Her-2 expression rates (*P*<0.001) compared with IDC-NSTs. Both features, along with low lymph node invasion frequency, may make PMBC a less aggressive cancer.

Global miRNA profiling of PMBC tissue and corresponding normal tissue identified differently expressed miRNAs using miRNA-microarray. We validated the expression profile in more patients by RT-qPCR and found that miR-143 and miR-224-5p were significantly downregulated in PMBC tissue. Results from immunohistochemical assay showed that PMBCs present significantly higher ER positive rate, lower HER-2 positive rate, and lower cell proliferation rate compared with IDC-NSTs. These findings will help us better understand the molecular mechanism underlying PMBC.

Several reports have demonstrated that PMBC shows a less aggressive behavior and a better clinical outcome than invasive ductal carcinoma^5^^,^^27^^-^^30^. Di Saverio *et al.*^2^ performed a retrospective review with long-term follow up on 11,400 cases of PMBC and found that positive nodal status is the most significant predictor of worse prognosis. Lacroix-Triki *et al.*^3^ analyzed the genomic alteration of PMBC and IDC-NST using array-comparative genomic hybridization technology and found that PMBCs are more homogenous and could be clustered together. These data are consistent with those of several previous reports^2^^,^^3^^,^^31^ and contribute in achieving better understanding of PMBC.
